# Antiproliferative and Antioxidant Properties of Anthocyanin Rich Extracts from Blueberry and Blackcurrant Juice

**DOI:** 10.3390/ijms16022352

**Published:** 2015-01-22

**Authors:** Zoriţa Diaconeasa, Loredana Leopold, Dumitriţa Rugină, Huseyin Ayvaz, Carmen Socaciu

**Affiliations:** 1 Faculty of Food Science and Technology, University of Agricultural Science and Veterinary Medicine Cluj-Napoca, Calea Mănăştur 3-5, Cluj-Napoca 400372, Romania; E-Mails: zorita.sconta@usamvcluj.ro (Z.D.); carmen.socaciu@usamvcluj.ro (C.S.); 2 Faculty of Veterinary Medicine, University of Agricultural Science and Veterinary Medicine Cluj-Napoca, Calea Mănăştur 3-5, Cluj-Napoca 400372, Romania; E-Mail: dumitrita.rugina@usamvcluj.ro; 3 Department of Food Engineering, Canakkale Onsekiz Mart University, Canakkale 17020, Turkey; E-Mail: ayvaz61@yahoo.com

**Keywords:** blueberry juice, blackcurrant juice, anthocyanins, antioxidant activity, cell viability, cancer

## Abstract

The present study was aimed at evaluating the antiproliferative potential of anthocyanin-rich fractions (ARFs) obtained from two commercially available juices (blueberry and blackcurrant juices) on three tumor cell lines; B16F10 (murine melanoma), A2780 (ovarian cancer) and HeLa (cervical cancer). Individual anthocyanin determination, identification and quantification were done using HPLC-MS. Antioxidant activity of the juices was determined through different mechanism methods such as DPPH and ORAC. For biological testing, the juices were purified through C_18_ cartridges in order to obtain fractions rich in anthocyanins. The major anthocyanins identified were glycosylated cyanidin derivatives. The antiproliferative activity of the fractions was tested using the MTT assay. The antiproliferative potential of ARF was found to be associated with those bioactive molecules, anthocyanins due to their antioxidant potential. The results obtained indicated that both blueberry and blackcurrants are rich sources of antioxidants including anthocyanins and therefore these fruits are highly recommended for daily consumption to prevent numerous degenerative diseases.

## 1. Introduction

Daily consumption of fruits and vegetables may have noticeable long-term physiological effects due to their bioactivity [1,2,3,4]. Apart from many bioactive compounds such as vitamins, minerals, sugars, organic acids, dietary fibres, berries and vegetables also contain phenolic compounds including flavonoids, tannins, stilbenoids, phenolic acids and lignans [5,6,7].

Being part of flavonoids, anthocyanins are the greatest natural pigments giving blue color in plants and showing antioxidant potential. The chemical structure of anthocyanins seems to be responsible for this great property [8,9]. Anthocyanins are the most oxidized flavonoids with the C ring fully unsaturated and a hydroxyl at position 3 [10,11,12]. They also can be found as glycosylated forms of polyhydroxy and polymethoxy derivatives of 2-phenylbenzopyrylium, acylated or not with aliphatic acids [13,14]. The richest sources of anthocyanins among many fruits and vegetables are the small berries like chokeberries, blueberries, elderberries and cranberries. Many researchers tested berry extracts against most common disorders and obtained significant results. It has been suggested that the consumption of colored fruits and vegetables are associated with reduced risk of human breast cancer [15,16], human melanoma cancer [17,18], and human ovarian cancer [19].

Blueberry fruits are the most commonly consumed berries in Romania. Belonging to genus *Vaccinum*, *fam. Ericaceae*, these fruits are known for their potential health benefits against degenerative diseases. Several studies showed that a rich diet in blueberries help to support arterial structure by helping maintaining healthy blood flow via LDL oxidation, normal platelet aggregation, and endothelial function improvement [20,21,22]. Matchett *et al.* [23] reported that blueberry flavonoids down-regulate the structure-degrading enzyme that enables cancerous cells to spread and invade other tissues. Blueberry extracts were also shown to inhibit the growth of tumor cells [24,25,26] and have anticarcinogenic properties [27]. The most common anthocyanidins found in wild and cultivated blueberries are cyanidin, delphinidin, petunidin, paeonidin, pelargonidin, and malvidin [28].

Another rich source of naturally occurring anthocyanins in plants is blackcurrants (*Ribes nigrum*, *fam. Grossulariaceae*). Originating in Northern Europe and Asia, these berries have been shown to have the potential to regulate and inhibit inflammation mechanisms suspected to trigger heart disease, cancer or microbial infections [29,30,31]. Major anthocyanins in blackcurrant are delphinidin-3-*O*-glucoside, delphinidin-3-*O*-rutinoside, cyanidin-3-*O*-glucoside, and cyanidin-3-*O*-rutinoside [32].

Our objective was to determine the total flavonoid, polyphenol content, antioxidant activity, anthocyanin profile and to evaluate the corresponding antiproliferative properties of ARFs obtained from blueberry and blackcurrant juice on several tumor cells lines.

## 2. Results and Discussion

In this study, the chemical compositions of two berry juices rich in anthocyanins (blueberry and blackcurrant) were compared. In total, 14 different compounds were purified, isolated, identified and they were further tested for their antiproliferative and apoptosis induction potential. Chemical characterization of blueberry anthocyanins using HPLC/PDA-MS disclosed that the major anthocyanins identified were malvidin-3-*O*-galactoside, petunidin-3-*O*-galactoside and delphinidin-3-*O*-galactoside. Blackcurrant purified ARFs were characterized by the presence of delphinidin-3-*O*-rutinoside as major anthocyanins.

### 2.1. Identification and Quantification of Anthocyanins

**Figure 1:**
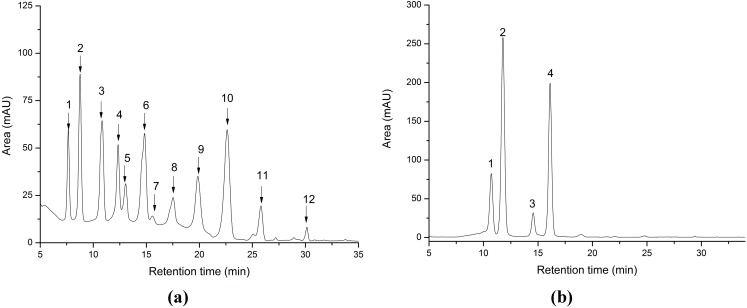
HPLC chromatograms of (**a**) blueberry *Vaccinum* sp., and (**b**) blackcurrant *Ribes nigrum* juices (Peaks assignments are shown in Table 1).

The anthocyanin identification was done based on their retention time, UV-VIS spectra and mass spectral analysis compared with standards and literature data [33,34,35,36]. This identification was strongly correlated with the studied reference values [35,37,38,39]. The absorbance was recorded at 520 nm (Figure 1). The chromatograms obtained revealed the presence of numerous anthocyanins and their derivates in the analyzed fruits juices. The peak identification, retention times, identification and their concentrations are summarized in Table 1.

Utilizing the HPLC–PDA chromatograms and MS spectra, 12 anthocyanins in total were identified in blueberry juice; with delphinidin and malvidin being the major anthocyanins. This was in accordance with recently published data [40,41]. On the other hand, purified ARFs obtained from blackcurrant juice was characterized by the presence of four individual anthocyanins (delphinidin-3-*O*-glucoside, delphinidin-3-*O*-rutinoside, cyanidin-3-*O*-glucoside, cyanidin-3-*O*-rutinoside in the order of elution) as previously reported [37,42], with cyanidin-3-*O*-glucoside being the major anthocyanin identified.

**Table 1:** Chromatographic characteristics and mass spectral data of anthocyanins obtained from blueberry and blackcurrant purified juices.

Sample Name	Peak No	Rt (min)	Peak Assignment	Concentration (mg/100mL) *	[M + H] + Ion/Fragments
Blueberry	1	8.1	Delphinidin-3-*O*-galactoside	11.46 ± 1.12	465/303
	2	9.3	Delphinidin-3-*O*-glucoside	22.63 ± 1.45	465/303
	3	11.2	Cyanidin-3-*O*-galactoside	6.42 ± 1.60	449/287
	4	11.5	Delphinidin-3-*O*-arabinoside	11.23 ± 1.93	435/303
	5	12.9	Cyanidin-3-*O*-glucoside	14.2 ± 1.86	449/287
	6	13.6	Petunidin-3-*O*-galactoside	6.33 ± 1.67	479/317
	7	15.2	Cyanidin-3-*O*-arabinoside	22.3 ± 1.54	419/287
	8	18.0	Paeonidin-3-*O*-galactoside	2.65 ± 1.04	463/301
	9	20.5	Petunidin-3-*O*-arabinoside	5.42 ± 1.33	449/317
	10	23.4	Malvidin-3-*O*-galactoside	10.66 ± 1.21	493/331
	11	26.3	Malvidin-3-*O*-glucoside	26 ± 1.96	493/331
	12	26.3	Malvidin-3-*O*-arabinoside	4.6 ± 2.06	463/331
Total				143.90 ± 1.56	
Blackcurrant	1	10.72	Delphinidin-3-*O*-glucoside	22.3 ± 1.36	465/303
	2	11.77	Delphinidin-3-*O*-rutinoside	57.95 ± 2.31	300/283/252
	3	14.8	Cyanidin-3-*O*-glucoside	9.9 ± 1.79	449/287
	4	16.01	Cyanidin-3-*O*- rutinoside	50.6 ± 1.63	595/287
Total				140.75 ± 1.77	

* Results are expressed as milligram of fraction equivalent (Cyanidin-3-*O*-rutinoside) per gram of mL sample (mean ± SD) (*n* = 3).

### 2.2. Total Monomeric Anthocyanins, Flavonoid and Total Polyphenol Content

The values obtained for anthocyanins, total flavonoid and polyphenol content of the purified juices are given in Table 2. These compounds are found in large amounts in fruits and contain active molecules with health benefits [43]. Total anthocyanin content obtained was 711.3 ± 0.82 mg and 580.4 ± 0.86 cyanidin-3-*O*-galactoside/100 mL for blueberry and blackcurrant juices, respectively. The results for total phenolic content of purified juices were lower than those of previously reported [44,45,46]. However, the values were in good correlation with data reported previously [39,47,48,49,50]. The differences could be attributed to the specific methods used during juice preparation. The results can be a confirmation that berry juices could be a good source of phenolic compounds. In order to determine significant differences between values, analysis of *t*-test was performed. Significance of difference was defined at the 5% level (*p* < 0.05).

**Table 2:** Total polyphenol, flavonoid and anthocyanin content in analyzed samples.

Sample	Total Polyphenol GAE mg/100 mL ^a^	Total Flavonoid QE mg/100 g ^b^	Total Anthocyanin C3GE mg/100 g ^c^
Blueberry	711.3 ± 0.82 ^#^	96.0 ± 0.96 ^#^	360.0 ± 0.76 ^#^
Blackcurrant	580.4 ± 0.86	84.6 ± 0.71	116.1 ± 0.59

^a^ Expressed in gallic acid equivalent (GAE), (mg GAE/100 g FW); ^b^ Expressed in quercetin equivalent, (mg/100 g FW); ^c^ Expressed in cyanidin-3-galactoside equivalent, (mg/100 g FW); # extremely significant, *p* < 0.001.

### 2.3. Antioxidant Activity

**Figure 2:**
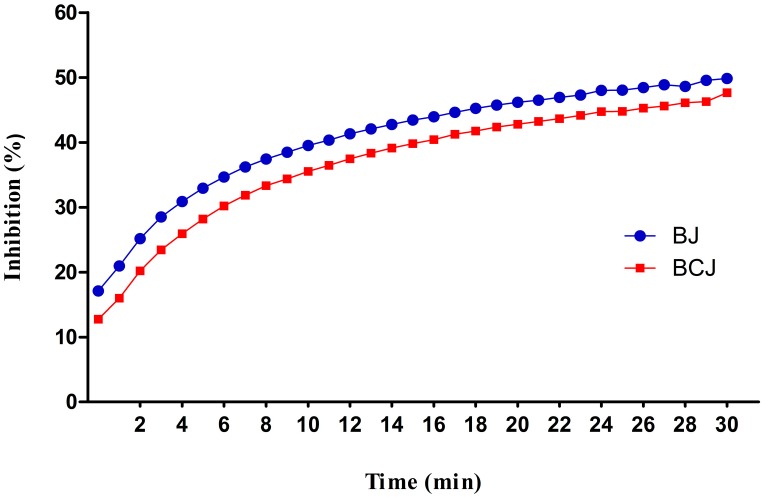
Antioxidant potency of blueberry (BJ) and blackcurrant (BCJ) juices using DPPH method. The inhibition percentage represents the antioxidant activity.

Literature data relieved a strong correlation between total polyphenols, antioxidant activity, and antitumoral protection of polyphenols’ intake [51]. For measuring the total antioxidant activity, DPPH and ORAC methods were used. The DPPH scavenging activity of analyzed samples was monitored kinetically for 30 min and presented in Figure 2. The results were in agreement with previously reported data [30,50]. The kinetic curves showed that differences among the analyzed samples regarding scavenging activity during 30 min were insignificant. Many studies reported antioxidant activity of fruits as ORAC values. This assay relieves the free-radical scavenging potential of antioxidants against peroxyl radical. For blueberry juice, 23.5 ± 0.23 μmol TE/mL was obtained, which was in agreement with those obtained by Seeram *et al.* [30] and Prior *et al.* [52] while lower than the values reported for rabbiteye blueberries by Wang *et al.* [53]. Regarding the ORAC values for blackcurrant juice, the results (19.39 ± 0.18 μmol TE/mL) were significantly lower than those reported by Hosseini *et al.* [54]. This finding could be attributed to different growth conditions of fruits and the methodology used to obtain the juice.

### 2.4. Analysis of Cell Proliferation

**Figure 3:**
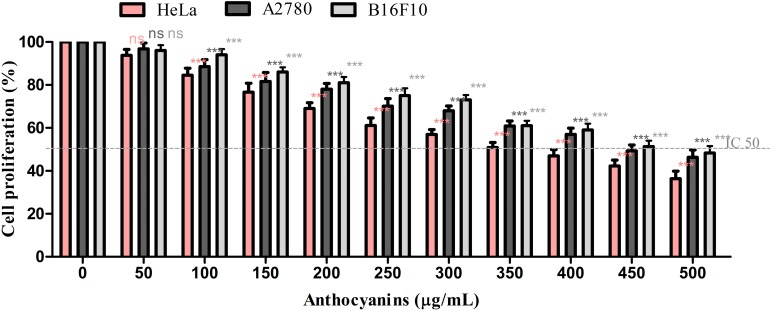
The antiproliferative effect of blueberry ARF on cancer cell lines. Statistical analysis was done using Dunnett multiple comparison test of Graph Pad Prism version 5.00. Data represent the means ± SD of at least three independent experiments (ns, none significant, *** extremely significant *p* < 0.001).

**Figure 4:**
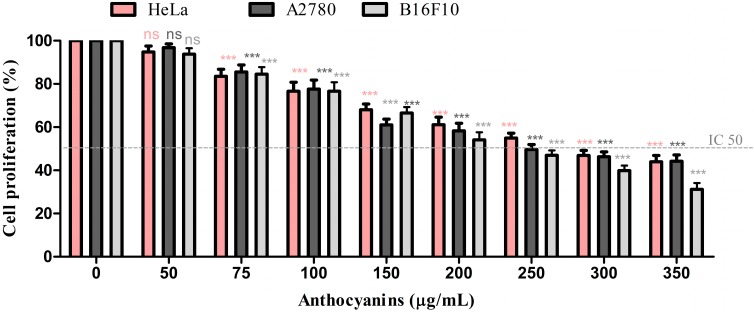
The antiproliferative effect of blackcurrant ARFs on cancer cell lines Statistical analysis was done using Dunnett multiple comparison test of Graph Pad Prism version 5.00. Data represent the means ± SD of at least three independent experiments (ns, none significant, *** extremely significant *p* < 0.001).

Purified ARFs obtained from blueberry and blackcurrant juices were evaluated for their antiproliferative proprieties on A2780, HeLa and B10F10 cancer lines. In order to evaluate the anti-proliferative effect of purified ARFs, the 3-(4,5)-dimethylthiazoly1-3,5-diphenyltetrazolium bromide (MTT) assay was used (Figure 3 and Figure 4).

For all cells lines, the treatment showed a dose dependent decrease of cell viability. Among the tested extracts, purified ARFs obtained from berry juices exhibited high antiproliferative activity against different tumor cell lines used. The treatment for 24 h with blueberry purified ARFs revealed that cervical cells were the most sensitive to purified ARFs. The cell viability was decreased from 100% to 45.9%, A2780 52% and 56% for HeLa, A2780 and B16F10, respectively, after 24 h exposure to blueberry purified ARFs (expresses as cyanidin 3-*O*-galactoside). Our results agreed with previous studies that reported high antiproliferative activity of ARFs from different berries [18,34,55,56]. Regarding the effects of anthocyanins of blueberry extract on cervical cancer, significant inhibition of cell growth was reported in a recent study following the 48 h treatment. [57]. Anthocyanin-rich extract obtained from açai remarkably suppressed proliferation of C-6 rat brain glioma cells, while no effect was found on the growth of MDA-468 human breast cancer cells. Additionally, the same experiment demonstrated that the AEA treatment inhibited the growth of C-6 rat glioma cells dose-dependently with an IC_50_ (the half maximal inhibitory concentration) of 121 μg/mL [33].

The glycosylated anthocyanins from blackcurrant showed a dose dependent decrease of cell viability against HeLa, A2780, and B16F10 cells with IC_50_ values of 281, 259.8 and 224 μg/mL, respectively (Figure 4). The results were similar with those reported by Olsson *et al.* [58]. Therefore, a concentration of 200, 250 and 300 μg/mL blackcurrant ARF for 24 h was chosen as standard treatment for the following experiments. Anthocyanin-rich black currant extract exhibited a potent cytotoxic effect on HepG2 cells [59].

An antiproliferative study conducted by Wu *et al.* [60] on B16F10 melanoma cells revealed that polyphenols obtained from pitaya peel and flesh (EC_50_ 25.0 μg of peel matter) inhibited the growth of B16F10 melanoma cancer cells. Similarly, recent studies showed that blackcurrant extracts had the potential to inhibit the growth of a number of cancer cell lines: HeLa, Fem X, LS 174, MCF-7 and PC-3 [47]. In another study [61], inhibition of HT-29 cancer cell proliferation due to blackcurrants was reported 0%–51% and for Caco-2 cancer cell proliferation, the range of inhibition was 10%–56%.

To best of our knowledge, there is no study available yet regarding the effects of blackcurrant anthocyanins on cell proliferation of murine melanoma cells. Natural supplements, a rich diet in antioxidants used as a complementary medication, become a common field of research in order to develop new products originating from natural sources with antioxidant and chemopreventive properties. Berry fruits such as blueberry, blackcurrant, and raspberries are some of the richest sources of phenolic bioactive compounds: flavonoids, anthocyanins, phenolic acids, which are well known for their chemopreventive or antiproliferative potential.

## 3. Experimental Section

### 3.1. Sample Preparation

Commercially available blueberry and blackcurrant juices were purchased from local market. The semipurification was done by solid-phase extraction (SPE) using C_18_ Sep-Pak cartridges following the procedure described by Giusti *el al*. [62]. Briefly, the samples were loaded into an activated C_18_ Sep-Pak cartridge (Waters, Milford, MA, USA). Sugars, acids, and other water-soluble compounds were removed by washing with 0.01% aqueous HCl. Then, polyphenols (other than anthocyanins) with EtOAc was removed. ARF was eluted and collected in acidified methanol to avoid their degradation. The purified extract obtained was filtered through 0.45 μm for further investigation. The methanol was removed at 35 °C under reduced pressure (Rotavapor R-124, Buchi, Switzerland). Following the purification, the obtained ARFs of the juices were dissolved in a known amount of sterile acidified water, filtered through 0.45 μm and then analyzed by liquid chromatography.

### 3.2. Quantification of Total Anthocyanins

Total anthocyanins were determined by the differential pH method based on the property of anthocyanins pigments to change their color as a response to changing pH. Two dilutions of the same sample were prepared; the first one in potassium chloride buffer (0.025 M, pH 1.0), and the second one in sodium acetate buffer (0.4 M, pH 4.5), with pH being adjusted using HCl. After equilibration at room temperature for 15 min, the absorbance of two dilutions was read at 510 and 700 nm by spectrophotometer (JASCO V-630 series, International Co., Ltd., Hachioji, Tokyo, Japan). Total monomeric anthocyanins (mg cyanidin 3-galactoside equivalent/1 mL juice) were calculated as follows:

Anthocyanins content (mg/L) = (A × MW × DF × 1000)/ε × L
(1)

where A = (A510 nm pH 1.0 − A700 nm pH 1.0) − (A510 nm pH 4.5 − A700 nm pH 4.5); MW = cyanidin 3-galactoside molecular weight (484.84); DF = dilution factor; ε = cyaniding 3-galactoside molar absorbtivity in methanol/ HCl (34300 M^−1^∙cm^−1^); L = cell path length (1 cm) [63].

### 3.3. HPLC-PDA/-ESI-MS Identification and Quantification of Anthocyanins

HPLC analysis was performed on a Shimadzu system equipped with a binary pump delivery system LC-20 AT (Prominence), a degasser DGU-20 A3 (Prominence), diodearray SPD-M20 A UV-VIS detector (DAD). For separation of anthocyanins, a Luna Phenomenex C-18 column (5 μm, 25 cm × 4.6 mm) was used. The mobile phases used were solvent A—formic acid (4.5%) in distilled water and solvent B—acetonitrile (100%). The gradient elution was: 10% B, 0–9 min; 12% B, 10–17 min; 25% B 18–30 min; 90% B, 31–50 min; 10% B, 51–55 min. The flow rate was 0.8 mL/min and the analyses were performed at 35 °C. The chromatogram was monitored at 520 nm. Anthocyanins quantification was performed using cyanidin 3-*O*-galactoside. Anthocyanins identification and peak assignments were done based on their retention times, UV-VIS spectra comparing with standards and literature data published. As a confirmation, the samples were analyzed by HPLC-ESI-MS. For HPLC/MS analysis, the HPLC apparatus was interfaced into a Quadrupole 6110 mass spectrometer (Agilent Technologies, Chelmsford, MA, USA) equipped with an ESI probe. Typical conditions for mass spectral analysis in positive ion electrospray mode for anthocyanins were: spray voltage of 3000 V, a nebulising pressure of 40.0 psi and a temperature of 100 °C. Data was collected in full scan mode over a mass range of 260–1000 *m*/*z*.

### 3.4. Total Flavonoid Content

For determination of the total flavonoid content of purified ARFs, a colorimetric method was used [64]. The samples were mixed with 300 μL NaNO2 (5%). The obtained mixture was incubated at room temperature for 5 min and then treated with 300 μL AlCl_3_ (10%). Afterwards, 2 mL of NaOH (1 N) was added. The flavonoid content was expressed as mg quercetin equivalents/100 mL juice. Each determination was carried out in triplicate.

### 3.5. DPPH-Scavenging Activity

The antioxidant activities of the analyzed samples were measured using the method from Brand-Williams *et al.* [65]. The reaction solution was prepared by allowing 35 μL sample and 250 μL of fresh prepared DPPH methanolic solution (80 μM) to react. The absorbance was then measured at 515 nm for 30 min. The antioxidant activity was calculated as follows:

DPPH scavenging effect (%) = [(A_0_ − A_s_) × 100]/A_0_ (2)

where, A_0_ is absorbance of the blank, and A_s_ is absorbance of the samples at 515 nm.

### 3.6. Oxygen Radical Absorbance Capacity (ORAC) Assay

The antioxidant capacity was also evaluated by the ORAC assay, following the protocol described previously [66]. The working solution was obtained by combining 150 μL sodium fluorescein solution (4 × 10^−3^ μM) in phosphate buffer (75 μM, pH 7.4). Then, 25 μL of each analyzed sample was added and incubated for 30 min at 37 °C. The reaction was initiated by the addition of 25 μL AAPH solution. The fluorescence was recorded kinetically for 30 min at 37 °C, at excitation wavelength of 485 ± 20 nm and emission wavelength of 525 ± 20 nm using a fluorescence microplate reader (BioTek Instruments, Winooski, VT, USA). ORAC values were expressed as μmol Trolox/1 mL juice.

### 3.7. Cell Culture

The A2780 human ovarian tumor cell line was maintained in RPMI medium supplemented with 10% FBS, 2 mM glutamine, 1% penicillin and streptomycin 0.1% as antibiotic. Human tumor cervical HeLa cell line was maintained in Dulbecco’s Modified Eagle Medium (DMEM) containing 1 g/L glucose supplemented with 10% fetal bovine serum, 1 mM glutamine, 1% gentamicin, 1% non-essential aminoacids. For B16F10 murine melanoma cells the grow media was consistent in Dulbecco’s Modified Eagle Medium (DMEM) containing 1 g/L, fetal bovine serum to a final concentration of 10%, 1 mM glutamine, 1% penicilin, streptomycin. Cell cultures were maintained at 37 °C in a humidified atmosphere of 5% relative humidity. HeLa and A2780 cells (5 × 10^3^ cells/well) were placed on 96-well microplate and treated after 24 h. B16F10 cells (8 × 10^3^ cells/well) were seeded on 96-well plate and cultured in DMEM containing 10% FBS for 24 h. The culture medium was then replaced with complete medium containing concentrations in the range of 0–500 μg/mL anthocyanins for blueberry purified ARFs and 0–350 μg/mL for blackcurrant purified ARFs. The stock solution was prepared with complete medium containing 0.3% DMSO.

### 3.8. Analysis of Cell Proliferation

Proliferation was measured using the MTT (dimethylthiazol diphenyl tetrazolium bromide) assay. After 24 h, the treated media was removed and MTT solution (0.5 mg/mL) was added to each well. After 2 h of incubation, MTT solution was removed and the formazan crystals were dissolved in DMSO. The solubilized formazan formed in viable cells was measured at 550 and 630 nm (for sample and background, respectively) using the microplate reader (HT BioTek Synergy, BioTek Instruments, Winooski, VT, USA). The results were expressed as percent survival relative to an untreated control. Each treatment was done in triplicate and repeated in five wells.

## 4. Conclusions

In this study, *in vitro* antiproliferative potential of ARF of blueberry and blackcurrant juices was demonstrated. The ARFs were analyzed for total flavonoid, polyphenol content and antioxidant activity. Moreover, HPLC-MS identification and quantification of individual anthocyanins was done. ARF were found to have strong antioxidant potential and inhibit cell proliferation on HeLa, A2780 and B16F10 tumor cells. However, there is currently no evidence that berry anthocyanins would significantly improve antioxidant status of organisms or the quality of life. In order to claim that blueberry or blackcurrant anthocyanins intake can reduce cancer risk, further studies are required to better understand their molecular pathways and also their metabolisms. Furthermore, this study contributes to the chemical characterization of two berry juices currently consumed in Romania, increasing the number of documented berry juices. Overall, all findings in this study comprise an important contribution to anthocyanin research in biological studies.

## References

[1] Steinmetz K.A., Potter J.D. (1996). Vegetables, fruit, and cancer prevention: A review. J. Am. Diet. Assoc..

[2] Philpott M., Lim C.C., Ferguson L. (2009). Dietary protection against free radicals: A case for multiple testing to establish structure-activity relationships for antioxidant potential of anthocyanic plant species. Int. J. Mol. Sci..

[3] Muselík J., García-Alonso M., Martín-López M., Žemlička M., Rivas-Gonzalo J. (2007). Measurement of antioxidant activity of wine catechins, procyanidins, anthocyanins and pyranoanthocyanins. Int. J. Mol. Sci..

[4] Li J., Jiang Y. (2007). Litchi flavonoids: Isolation, identification and biological activity. Molecules.

[5] Liu R.H. (2013). Dietary bioactive compounds and their health implications. J. Food Sci..

[6] Liu R.H. (2013). Health-promoting components of fruits and vegetables in the diet. Adv. Nutr..

[7] Slavin J.L., Lloyd B. (2012). Health benefits of fruits and vegetables. Adv. Nutr..

[8] Wang H., Cao G., Prior R.L. (1997). Oxygen radical absorbing capacity of anthocyanins. J. Agric. Food Chem..

[9] He J., Giusti M.M. (2011). High-purity isolation of anthocyanins mixtures from fruits and vegetables—A novel solid-phase extraction method using mixed mode cation-exchange chromatography. J. Chromatogr. A.

[10] Gehm B.D., McAndrews J.M., Chien P.Y., Jameson J.L. (1997). Resveratrol, a polyphenolic compound found in grapes and wine, is an agonist for the estrogen receptor. Proc. Natl. Acad. Sci. USA.

[11] Welch C.R., Wu Q., Simon J.E. (2008). Recent advances in anthocyanin analysis and characterization. Curr. Anal. Chem..

[12] Brouillard R., Markakis P. (1982). Chapter 1—chemical structure of anthocyanins. Anthocyanins as Food Colors.

[13] Bochi V.C., Barcia M.T., Rodrigues D., Speroni C.S., Giusti M.M., Godoy H.T. (2014). Polyphenol extraction optimisation from ceylon gooseberry (dovyalis hebecarpa) pulp. Food Chem..

[14] He F., Mu L., Yan G.L., Liang N.N., Pan Q.H., Wang J., Reeves M.J., Duan C.Q. (2010). Biosynthesis of anthocyanins and their regulation in colored grapes. Molecules.

[15] Adlercreutz H. (1998). Epidemiology of phytoestrogens. Baillieres Clin. Endocrinol. Metab..

[16] Fung T.T., Chiuve S.E., Willett W.C., Hankinson S.E., Hu F.B., Holmes M.D. (2013). Intake of specific fruits and vegetables in relation to risk of estrogen receptor-negative breast cancer among postmenopausal women. Breast Cancer Res. Treat..

[17] Serafino A., Sinibaldi-Vallebona P., Lazzarino G., Tavazzi B., Rasi G., Pierimarchi P., Andreola F., Moroni G., Galvano G., Galvano F. (2004). Differentiation of human melanoma cells induced by cyanidin-3-o-beta-glucopyranoside. FASEB J..

[18] Huang H.P., Shih Y.W., Chang Y.C., Hung C.N., Wang C.J. (2008). Chemoinhibitory effect of mulberry anthocyanins on melanoma metastasis involved in the ras/pi3k pathway. J. Agric. Food Chem..

[19] Akim A.M., Ling L.C., Rahmat A., Zakaria Z.A. (2011). Antioxidant and anti-proliferative activities of roselle juice on caov-3, mcf-7, mda-mb-231 and hela cancer cell lines. Afr. J. Pharm. Pharmacol..

[20] Shaughnessy K.S., Boswall I.A., Scanlan A.P., Gottschall-Pass K.T., Sweeney M.I. (2009). Diets containing blueberry extract lower blood pressure in spontaneously hypertensive stroke-prone rats. Nutr. Res..

[21] Kalt W., Foote K., Fillmore S.A., Lyon M., Van Lunen T.A., McRae K.B. (2008). Effect of blueberry feeding on plasma lipids in pigs. Br. J. Nutr..

[22] De Pascual-Teresa S., Moreno D.A., García-Viguera C. (2010). Flavanols and anthocyanins in cardiovascular health: A review of current evidence. Int. J. Mol. Sci..

[23] Matchett M.D., MacKinnon S.L., Sweeney M.I., Gottschall-Pass K.T., Hurta R.A. (2006). Inhibition of matrix metalloproteinase activity in du145 human prostate cancer cells by flavonoids from lowbush blueberry (vaccinium angustifolium): Possible roles for protein kinase c and mitogen-activated protein-kinase-mediated events. J. Nutr. Biochem..

[24] Liu J., Zhang W., Jing H., Popovich D.G. (2010). Bog bilberry (vaccinium uliginosum l.) extract reduces cultured hep-g2, caco-2, and 3t3-l1 cell viability, affects cell cycle progression, and has variable effects on membrane permeability. J. Food Sci..

[25] Yi W., Fischer J., Krewer G., Akoh C.C. (2005). Phenolic compounds from blueberries can inhibit colon cancer cell proliferation and induce apoptosis. J. Agric. Food Chem..

[26] Liu W., Lu X., He G., Gao X., Xu M., Zhang J., Li M., Wang L., Li Z., Wang L. (2013). Protective roles of gadd45 and mdm2 in blueberry anthocyanins mediated DNA repair of fragmented and non-fragmented DNA damage in uv-irradiated hepg2 cells. Int. J. Mol. Sci..

[27] Stoner G.D., Wang L.S., Casto B.C. (2008). Laboratory and clinical studies of cancer chemoprevention by antioxidants in berries. Carcinogenesis.

[28] Veberic R., Slatnar A., Bizjak J., Stampar F., Mikulic-Petkovsek M. (2015). Anthocyanin composition of different wild and cultivated berry species. LWT Food Sci. Technol..

[29] Heinonen M. (2007). Antioxidant activity and antimicrobial effect of berry phenolics—A finnish perspective. Mol. Nutr. Food Res..

[30] Seeram N.P. (2008). Berry fruits: Compositional elements, biochemical activities, and the impact of their intake on human health, performance, and disease. J. Agric. Food Chem..

[31] Bonarska-Kujawa D., Cyboran S., Żyłka R., Oszmiański J., Kleszczyńska H. (2014). Biological activity of blackcurrant extracts (ribes nigrum l.) in relation to erythrocyte membranes. Biomed Res. Int..

[32] Kapasakalidis P.G., Rastall R.A., Gordon M.H. (2006). Extraction of polyphenols from processed black currant (ribes nigrum l.) residues. J. Agric. Food Chem..

[33] Hogan S., Chung H., Zhang L., Li J., Lee Y., Dai Y., Zhou K. (2010). Antiproliferative and antioxidant properties of anthocyanin-rich extract from açai. Food Chem..

[34] Zhang Z., Knobloch T.J., Seamon L.G., Stoner G.D., Cohn D.E., Paskett E.D., Fowler J.M., Weghorst C.M. (2011). A black raspberry extract inhibits proliferation and regulates apoptosis in cervical cancer cells. Gynecol. Oncol..

[35] Rubinskiene M., Jasutiene I., Venskutonis P.R., Viskelis P. (2005). Hplc determination of the composition and stability of blackcurrant anthocyanins. J. Chromatogr. Sci..

[36] Bordonaba J.G., Crespo P., Terry L.A. (2011). A new acetonitrile-free mobile phase for hplc-dad determination of individual anthocyanins in blackcurrant and strawberry fruits: A comparison and validation study. Food Chem..

[37] J. Bermúdez-Soto M., A. Tomás-Barberán F. (2004). Evaluation of commercial red fruit juice concentrates as ingredients for antioxidant functional juices. Eur. Food Res. Technol..

[38] Goiffon J.P., Brun M., Bourrier M.J. (1991). High-performance liquid chromatography of red fruit anthocyanins. J. Chromatogr. A.

[39] Jakobek L., Šeruga M., Medvidović-Kosanović M., Novak I. (2007). Antioxidant activity and polyphenols of aronia in comparison to other berry species. ACS.

[40] Bunea A., Rugina D., Sconta Z., Pop R.M., Pintea A., Socaciu C., Tabaran F., Grootaert C., Struijs K., VanCamp J. (2013). Anthocyanin determination in blueberry extracts from various cultivars and their antiproliferative and apoptotic properties in b16-f10 metastatic murine melanoma cells. Phytochemistry.

[41] Wu X., Beecher G.R., Holden J.M., Haytowitz D.B., Gebhardt S.E., Prior R.L. (2006). Concentrations of anthocyanins in common foods in the united states and estimation of normal consumption. J. Agric. Food Chem..

[42] Nielsen I.L., Haren G.R., Magnussen E.L., Dragsted L.O., Rasmussen S.E. (2003). Quantification of anthocyanins in commercial black currant juices by simple high-performance liquid chromatography. Investigation of their pH stability and antioxidative potency. J. Agric. Food Chem..

[43] Clifford M.N. (2000). Anthocyanins—nature, occurrence and dietary burden. J. Sci. Food Agric..

[44] Costantino L., Albasini A., Rastelli G., Benvenuti S. (1992). Activity of polyphenolic crude extracts as scavengers of superoxide radicals and inhibitors of xanthine oxidase. Planta Med..

[45] Rotundo A., Bounous G., Benvenuti S., Vampa G., Melegari M., Soragni F. (1998). Quality and yield of ribes and rubus cultivars grown in southern italy hilly locations. Phytother. Res..

[46] Moyer R.A., Hummer K.E., Finn C.E., Frei B., Wrolstad R.E. (2002). Anthocyanins, phenolics, and antioxidant capacity in diverse small fruits: Vaccinium, rubus, and ribes. J. Agric. Food Chem..

[47] Konić-Ristić A., Šavikin K., Zdunić G., Janković T., Juranic Z., Menković N., Stanković I. (2011). Biological activity and chemical composition of different berry juices. Food Chem..

[48] Mattila P.H., Hellström J., McDougall G., Dobson G., Pihlava J.M., Tiirikka T., Stewart D., Karjalainen R. (2011). Polyphenol and vitamin C contents in european commercial blackcurrant juice products. Food Chem..

[49] Benvenuti S., Pellati F., Melegari M., Bertelli D. (2004). Polyphenols, anthocyanins, ascorbic acid, and radical scavenging activity of rubus, ribes, and aronia. J. Food Sci..

[50] Piljac-Žegarac J., Valek L., Martinez S., Belščak A. (2009). Fluctuations in the phenolic content and antioxidant capacity of dark fruit juices in refrigerated storage. Food Chem..

[51] Kai H., Fuse T., Kunitake H., Morishita K., Matsuno K. (2014). Comparison of cultivars and seasonal variation in blueberry (vaccinium species) leaf extract on adult t-cell leukemia cell line growth suppression. Medicines.

[52] Prior R.L., Cao G., Martin A., Sofic E., McEwen J., O’Brien C., Lischner N., Ehlenfeldt M., Kalt W., Krewer G. (1998). Antioxidant capacity as influenced by total phenolic and anthocyanin content, maturity, and variety of vaccinium species. J. Agric. Food Chem..

[53] Wang C.Y., Chen C.T., Wang S.Y. (2009). Changes of flavonoid content and antioxidant capacity in blueberries after illumination with uv-c. Food Chem..

[54] Hosseini-Beheshti E., Lund S.T., Kitts D.D. (2012). Characterization of antioxidant capacity from fruits with distinct anthocyanin biosynthetic pathways. Nutr. Food Sci..

[55] Zhang Y., Seeram N.P., Lee R., Feng L., Heber D. (2008). Isolation and identification of strawberry phenolics with antioxidant and human cancer cell antiproliferative properties. J. Agric. Food Chem..

[56] Seeram N.P., Adams L.S., Zhang Y., Lee R., Sand D., Scheuller H.S., Heber D. (2006). Blackberry, black raspberry, blueberry, cranberry, red raspberry, and strawberry extracts inhibit growth and stimulate apoptosis of human cancer cells *in vitro*. J. Agric. Food Chem..

[57] Jiang Y.X., Lei J.T., LV Shi J. (2010). Bilberry extract anthocyanins on effect of cervical cancer hela cells. Matern Child Health J..

[58] Olsson M.E., Gustavsson K.E., Andersson S., Nilsson A., Duan R.D. (2004). Inhibition of cancer cell proliferation *in vitro* by fruit and berry extracts and correlations with antioxidant levels. J. Agric. Food Chem..

[59] Bishayee A., Haznagy-Radnai E., Mbimba T., Sipos P., Morazzoni P., Darvesh A.S., Bhatia D., Hohmann J. (2010). Anthocyanin-rich black currant extract suppresses the growth of human hepatocellular carcinoma cells. Nat. Prod. Commun..

[60] Wu L.C., Hsu H.W., Chen Y.C., Chiu C.C., Lin Y.I., Ho J.A.A. (2006). Antioxidant and antiproliferative activities of red pitaya. Food Chem..

[61] Khoo G.M., Clausen M.R., Pedersen H.L., Larsen E. (2012). Bioactivity and chemical composition of blackcurrant (ribes nigrum) cultivars with and without pesticide treatment. Food Chem..

[62] Giusti M.M., Rodriguez-Saona L.E., Wrolstad R.E. (1999). Molar absorptivity and color characteristics of acylated and non-acylated pelargonidin-based anthocyanins. J. Agric. Food Chem..

[63] Giusti M.M., Wrolstad R.E. (2001). Characterization and measurement of anthocyanins by uv-visible spectroscopy. Current Protocols in Food Analytical Chemistry.

[64] Zhishen J., Mengcheng T., Jianming W. (1999). The determination of flavonoid contents in mulberry and their scavenging effects on superoxide radicals. Food Chem..

[65] Brand-Williams W., Cuvelier M.E., Berset C. (1995). Use of a free radical method to evaluate antioxidant activity. LWT Food Sci. Technol..

[66] Huang D., Ou B., Prior R.L. (2005). The chemistry behind antioxidant capacity assays. J. Agric. Food Chem..

